# The pathophyiological role of aminoacyl-tRNA synthetases in digestive system diseases

**DOI:** 10.3389/fphys.2022.935576

**Published:** 2022-08-09

**Authors:** Wugelanmu Wusiman, Zerui Zhang, Qiang Ding, Mei Liu

**Affiliations:** ^1^ Department of Gastroenterology, Institute of Liver and Gastrointestinal Diseases, Tongji Hospital of Tongji Medical College, Huazhong University of Science and Technology, Wuhan, China; ^2^ Department of Gastroenterology, Tongji Hospital of Tongji Medical College, Huazhong University of Science and Technology, Wuhan, Hubei, China

**Keywords:** aminoacyl-tRNA synthetases, pathophyiology, malfunction, tumorigenesis, digestive system diseases

## Abstract

Aminoacyl-tRNA synthetases (ARSs) catalyze the ligation of amino acids to their cognate transfer RNAs and are indispensable enzymes for protein biosynthesis in all the cells. Previously, ARSs were considered simply as housekeeping enzymes, however, they are now known to be involved in a variety of physiological and pathological processes, such as tumorigenesis, angiogenesis, and immune response. In this review, we summarize the role of ARSs in the digestive system, including the esophagus, stomach, small intestine, colon, as well as the auxiliary organs such as the pancreas, liver, and the gallbladder. Furthermore, we specifically focus on the diagnostic and prognostic value of ARSs in cancers, aiming to provide new insights into the pathophysiological implications of ARSs in tumorigenesis.

## Introduction

Aminoacyl-tRNA synthetases (ARSs), which consist of 20 different enzymes, play a major role in protein synthesis. The catalytic reaction of ARSs consists of two steps. In the first step, the amino acid is activated by ATP and binds to its specific ARS to form an intermediate known as the aminoacyl-AMP. In the second step, the intermediate transfers the amino acid to the 3′-terminal adenosine of its cognate tRNA and releases AMP (Kwon et al., 2019). Subsequently, the aminoacylated tRNA is recruited to the ribosome, where it participates in protein synthesis. By consuming one molecule of ATP, ARSs ligate the appropriate amino acids to their cognate tRNAs with high fidelity, not only due to their accuracy in recognizing the correct substrate amino acids and the cognate tRNAs but also due to their special proofreading activity, which happens both before and after the ligation of the amino acid to the cognate tRNAs (Kim et al., 2011). The correct aminoacylation of tRNAs is critical for maintaining cellular viability. When there is an unmatched link between the amino acid and tRNA, ARSs hydrolyze the mischarged aa-tRNA complex.

In human cells, both the cytoplasm and the mitochondria have their specific ARSs. Each ARS protein and its encoding gene are identified as XRS and XARS, respectively, where X designates the amino acid, and for the mitochondrial ARSs, a “2” is appended. For example, LRS denotes the leucyl-tRNA synthetase and LARS is the gene encoding it, while LRS2 represents the mitochondrial leucyl-tRNA synthetase (Yao and Fox, 2013). There are a total of 19 ARS polypeptides in the human cytoplasm, performing the activity of 20 ARSs. The glutamyl-tRNA synthetase (ERS) and prolyl-tRNA synthetase (PRS) are naturally fused into a bifunctional glutamyl-prolyl-tRNA synthetase (EPRS) encoded by a single gene. As for the mitochondria, it encodes 17 unique ARSs proteins, with the addition of two dual localized ARSs, namely, the lysyl-tRNA synthetase (KRS) and the glycyl-tRNA synthetase (GRS). In the mitochondria, due to the lack of a functional glutaminyl tRNA synthetase (QRS), the ERS misacylates tRNA^Gln^ to form Glu-tRNA^Gln^, which is then transamidated by the amidotransferase to form Gln-tRNA^Gln^ (Nagao et al., 2009).

Despite their common functions, the family of ARSs belong to quite diverse groups. Based on their structural differences, the ARSs are classified into two classes (class I and class II), and each class consists of ten members and are further divided into three subclasses (a, b, and c), based on the chemical properties of the amino acids they recognize. One of the main difference between the two classes of ARSs is that the class I ARSs contain a characteristic Rossmann fold catalytic domain, while the Class II ARSs share a seven-stranded β-sheet. In class I synthetases, the Rossmann fold, which takes a nucleotide-binding role, contains the two consensus sequences: HIGH (His-Ile-Gly-His) and KMSKS (Lys-Met-Ser-Lys-Ser). The HIGH motif itself is located in the N-terminal half of the Rossmann fold, whereas the KMSKS motif is present in a loop in the second half of the fold. The HIGH motif correctly positions the ATP in the first step of the catalytic reaction, while the KMSKS motif recognizes and binds to the amino acids and stabilizes the aminoacyl-adenylate intermediate during amino acid activation. In class II ARSs, the β-sheet is characterized by three motifs known as motifs 1, 2, and 3. Motif 1 participates in dimer formation, and motifs 2 and 3 are involved in the formation of the aminoacyl-adenylate intermediate and binding to the 3′end of the tRNA. The other structural difference between the two enzyme classes is that class I ARSs are usually monomeric, while class II ARSs are dimeric or oligomeric (Kwon et al., 2019). Additionally, class I and class II ARSs also have differences in the second step of the catalytic reaction. The class I enzymes attach the activated amino acids to the 2′-hydroxyl of the tRNA acceptor stem, whereas attachment to the 3′-hydroxyl is specific for class II enzymes. Phenylalanyl-tRNA synthetase (FRS) is an exception to this rule. Although it is a class II synthetase, it ligates aminoacyl to the 2′-hydroxyl. Moreover, the class I ARSs approach the acceptor stem of the tRNA from the minor groove side, while the class II ARSs approach it from the major groove side (Beuning and Musier-Forsyth, 1999).

In mammals, ARSs function in a free form or a complex-bound form. The latter form, known as the multi-tRNA synthetase complex (MSC), includes eight different ARSs (EPRS, IRS, LRS, QRS, KRS, RRS, DRS, and MRS) and three ARS-interacting multifunctional proteins (AIMP1, AIMP2, and AIMP3). MSC harbors the component synthetases until they reach the target position where they come into play. The gathering of MSC is believed to increase the stability of its components, while it may dissociate due to posttranslational modifications (Han et al., 2006). AIMPs not only have a scaffolding role in the formation of MSC, but also have other cellular functions such as being a part of signaling pathways (Park S. G. et al., 2010).

As one of the oldest proteins, ARSs exist and catalyze the aminoacylation reaction in all protein-making cells and organelles, including the mitochondria, chloroplast, and apicoplast (Fang and Guo, 2015). Over the course of eukaryotic evolution, the ARSs have developed domains and motifs which are not essential for their catalytic activities, but their addition has increased the complexity of eukaryotes and has expanded the function of ARSs (Guo et al., 2010). Although there are common motifs across different ARSs, some motifs are unique to a specific aminoacyl synthetase.

**FIGURE 1:**
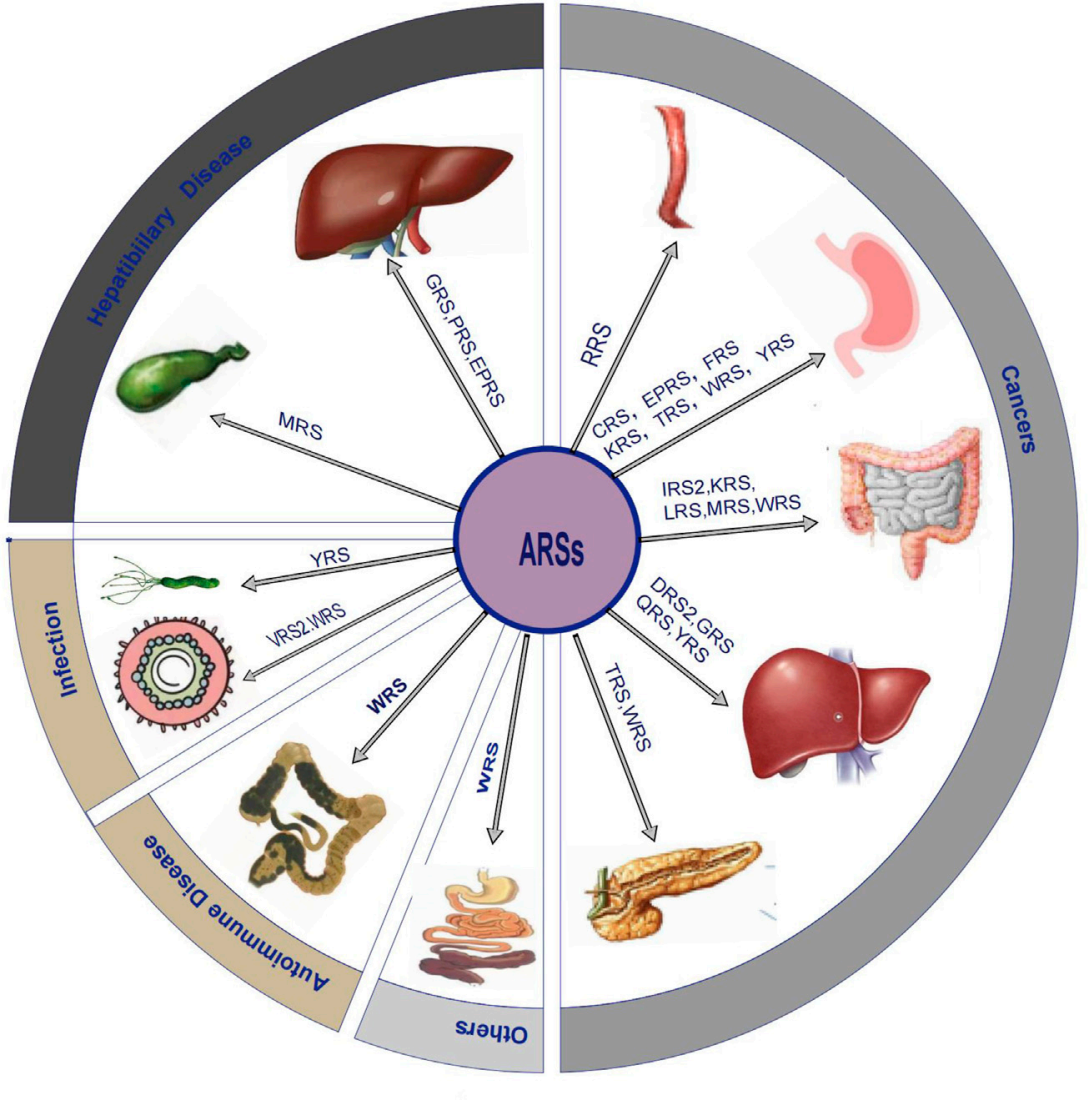
Overview diagram that shows ARSs related digestive system diseases.

An accumulating body of evidence suggests that the altered structure or expression of ARSs are related to pathological processes such as cancers, autoimmune diseases, and rare diseases. For instance, LRS, EPRS, and mitochondrial isoleucyl-tRNA synthetase (IRS2) are more abundant in different types of cancers (Katsyv et al., 2016; Wang et al., 2018; Li et al., 2019). As suggested by some of the earlier studies, Charcot Marie Tooth disease (CMT), which is regarded as an incurable neurodegenerative disease, is related to the mutation of several different members of the ARSs family, including GRS (Antonellis et al., 2003), tyrosyl-tRNA synthetase (YRS) (Jordanova et al., 2006), alanyl-tRNA synthetase (AlaRS) (Weterman et al., 2018), methionyl-tRNA synthetase (MRS) (Gonzalez et al., 2013), tryptophanyl-tRNA synthetase (WRS) (Tsai et al., 2017), and histidyl-tRNA synthetase (HRS) (Vester et al., 2013). Some clinical studies have shown that AlaRS2 was correlated with cardiomyopathy (Mazurova et al., 2017). Nevertheless, there are only a few reviews that elaborate on the role of ARSs in the digestive system. In the current review, we aimed to focus on the potential pathophysiological significance of ARSs in digestive system diseases, including cancer, and benign diseases such as hepatobiliary diseases, digestive system infection, autoimmune diseases, and others. We also displayed the information of this review in the form of picture and table (Figure 1; Table1).

**TABLE 1:** ARSs and their pathological impact.

ARSs	Organs	Disease	Physiological (mal)function	References
CRS	Stomach	Gastric cancer	SNPs in CARS influence the occurrence and development of gastric cancer	Tian et al. (2017)
DRS2	Liver	Hepatocellular carcinoma	Upregulated	Qin et al. (2017)
			Correlates with bigger tumor size, worse cell differentiation, distal metastasis, portal vein invasion, and shorter overall survival time	
			Accelerate cell cycle	
			Attenuate cell apoptosis	
		HBV infection	Upregulated in HBV-infected patients and HBV carrying cells	
EPRS	Stomach	Gastric cancer	Upregulated	Liu et al. (2021)
			Related to poor overall survival	
			EPRS knockdown inhibits cell prolifration and tumor growth	
	Liver	Liver fibrosis	Knockdown of EPRS inhibits the production, deposition, and transcriptional induction of ECMs	Song et al. (2019)
GRS	Liver	CCl4 induced hepatic damage	Secreted to peripheral circulation	Park et al. (2018)
			Mitigates liver and spleen enlargements	
		Liver fibrosis animal model	Enhance the recruitment of MSCs to the injury site	
			Stimulate the migration of MSCs	
IRS2	Colon	Colon cancer	Upregulated	Zhong et al. (2015)
			Knockdown of IRS2 inhibits the proliferation and clone formation, increases apoptosis	
KRS	Stomach	Gastric cancer	Upregulated in tumor cells and TAIs	Kim B. H. et al. (2014)
			Correlates with bigger tumor size, higher proliferation index, lymphovascular invasion, and shorter overall survival time	
	Colon	Colon cancer	Upregulated	Suh et al. (2020)
			Suppression of KRS causes incomplete EMT and impairs focal adhesion formation	Nam et al. (2016)
			Polarization of M1 macrophages into M2 macrophages	Nam et al. (2018)
MRS	Colon	Colon cancer	Upregulated	Kushner et al. (1976)
			Related to increased CRC risk in humans	Wang et al. (2018)
		HF dieted mouse and rat model	Upregulated	
			Induce DNA damage accumulation	
	Bile duct	Bile duct stricture	IHC staining of MRS used to distinguish benign and malignant bile duct strictures	Singhi and Slivka (2020), Jang et al. (2021)
PRS	Liver	liver cirrhosis	Higher in liver cirrhotic patients than in non-cirrhotic patients	Kershenobich et al. (1970)
QRS	Liver	Hepatocellular carcinoma	Suppress glutamine-related apoptosis	Xu et al. (1997), Ko et al. (2001)
RRS	Esophagus	Esophageal adenocarcinoma	Upregulated	Kang et al. (2020)
			Have specificity and sensitivity in assessing prognostic outcome of EAC	
TRS	Pancreas	Pancreatic cancer	Correlated with the poor survival	Jeong et al. (2018)
			Regulates MUC1 biosynthesis	
			Affect MUC1-mediated cancer cell migration	
VRS2	Liver	Hepatitis B	Varient of VRS2 associated with the risk of CHB	Cheong et al. (2015)
WRS	Stomach	Gastric cancer	Upregulated in EBV and MSI-H tumors	Lu et al. (2020)
			Associated with poor prognosis in p53 aberrant, p53-wildtype tumors	
			Associated with better prognosis in MSI tumors	
			Stimulated by IDO	
		Gastric cancer treated with adjuvant chemotherapy	Related to immune regulation and inflammatory response	Cheong et al. (2018)
	Intestine	GIST	Related to tumor size, mitoses, and outcomes	Blakely et al. (2018)
	Colon	IBD model (DSS-induced experimental colitis)	Downregulated	Kang et al. (2019)
			Upregulated by addition of hUCB-MSCs	
			Inhibits the proliferation of hUCB-MCSs derived CD4^+^ cells	
		Colon cancer	Upregualted and improves prognosis	Ghanipour et al. (2009)
	Pancreas	Pancreatic cancer	Full-length WRS is downregualted and T2-TrpRS upregulated in hypoxia	Paley et al. (2011)
	Liver	HBV infection	Associated with HBV replication-induced angiogenesis	Zhang et al. (2009)
YRS	Stomach	Gastric cancer	Upregulated	Zhang et al. (2020)
			Enhances proliferation and migration/invasion	
			Inhubits apoptosis	
			Induced enhancements of homologous recombination	

## Aminoacyl-tRNA synthetases and cancer

Recently, Hanahan (2022) elaborated the eight hallmarks of cancer, including capabilities for sustaining proliferative signaling, evading growth suppressors, resisting cell death, enabling replicative immortality, inducing/accessing vasculature, activating invasion and metastasis, reprogramming cellular metabolism, and avoiding immune destruction. Each of the characteristics of cancers mentioned above involves the precise regulation of innumerable molecules, which causes complexities in cancer pathogenesis as well as difficulties in its treatment. ARSs have been reported to participate in pathways that promote or suppress tumorigenesis. In this part of the review, we concentrate on the critical role of ARSs in the development of malignant tumours in the digestive system, including esophageal cancer, gastric cancer, colorectal cancer, liver cancer, and pancreatic cancer.

### Esophageal adenocarcinoma

Esophageal adenocarcinoma (EAC) is one of the most aggressive cancers in the world with a 5-year survival rate of just under 20%. One of its premalignant condition is Barrett’s esophagus, which is closely associated with the high malignancy of EAC. According to the publicly available datasets on GSEA (Gene set enrichment analysis), the expression level of RRS (Arginyl-tRNA synthetase) was upregulated in EAC tissues, as compared to the normal tissues and was associated with a poor prognosis (Kang et al., 2020). RARS also has been identified as a glycolysis-related gene, and the glycolysis pathway is known to be significantly upregulated in Barrett’s esophagus as compared to the normal squamous esophagus and gastric cardia (van Baal et al., 2006). Yet another effect of RARS on cancers involves gene fusion between RARS and mitotic arrest deficient 1-like protein 1 (MAD1L1), which induces the proliferation of nasopharyngeal cancer cell lines and their chemo- and radio-resistance (Zhong et al., 2018). Although the role of ARSs in the onset of EAC has not been studied in depth, the studies above indicated that ARSs, especially RRS, might play a potential role during the development of EAC from Barrett’s esophagus.

### Gastric cancer

Gastric cancer (GC) is the fifth most frequently diagnosed cancer and the third leading cause of cancer death worldwide. Despite the major efforts that have been made over the past decades, the prognosis of GC still remains poor because of the limited availability of therapeutic drug targets and the high tumor heterogeneity (Bray et al., 2018). An earlier study reported that proline and serine metabolism played an important role in GC metastasis (Chen et al., 2010). According to a study based on the metabolomic and transcriptomic analysis of 16 gastric cancer samples, the aminoacyl-tRNA pathway was upregulated in the cancer tissues in comparison to the adjacent non-cancerous tissues, and threonyl-tRNA synthetase (TRS) and phenylalanyl-tRNA synthetase (FRS) were correlated with tumor metastasis and worse prognosis (Gao et al., 2021). Studies conducted in the Chinese population reported that SNPs (single nucleotide polymorphisms) in the CARS (cysteinyl transfer RNA synthetase) gene influenced the biological functions of CARS as well as the onset and development of GC (Tian et al., 2017). Other ARSs that have effect on development of GC are highlighted in following text.

WRS increases the production of tryptophan (Trp), while indoleamine 2,3-dioxygenase (IDO) promotes the degradation of Trp. The balance between the above two enzymes decides the fate of Trp. Studies have shown that IDO1 and WRS were upregulated in EBV-associated and MSI-H gastric tumors and had prognostic significance in p53 mutant, as well as p53-wildtype tumors (Patil et al., 2018; Lu et al., 2020). IDO1 activity accelerates Trp depletion and upregulates the expression of WRS through the general control non-derepressible-2 kinase (GCN2)/phosphorylated eukaryotic translation initiation factor 2α (peIF2α)/activating transcription factor 4 (ATF4) signaling pathway (Adam et al., 2018). High expression of intratumoral IDO is significantly associated with the depth of tumor invasion and lymph node metastasis, as well as poor postoperative clinical outcome in GC patients (Liu et al., 2016). In dendritic cells (DC), Kupffer cells (KC), and macrophages, the expression of IDO inhibits T-cells proliferation (Munn et al., 1999; Hwu et al., 2000; Yan et al., 2010), thereby helping cancer cells to escape from immune surveillance. Besides, the upregulation of WRS produces more Trp, which nourishes the cancer cells. Although the precise mechanism by which IDO1 expression in macrophages, KC and DC suppresses CD8+ T cells, and the role of WRS in the development of GC are not fully understood, both WRS and IDO have been reported to play important roles in GC progression. The pivotal role of WRS in gastric tumorigenesis was also demonstrated in another comparative study which claimed that WRS was related to immune regulation and inflammatory response in resectable gastric cancer patients treated with adjuvant chemotherapy (Cheong et al., 2018).

YRS is highly expressed in all gastric cancer Lauren subtypes, based on public datasets such as Oncomine and TCGA-GC. Moreover, the IHC (immunohistochemical staining) data from 14 pairs of cancer and matched normal tissues also showed evidence of YRS’s higher expression in GC than in the normal tissues. Low expression of YRS inhibited cell proliferation, migration and invasion, but enhanced apoptosis. Consistent with the findings from *in vitro* experiments, high expression of YRS had significantly enhanced the tumor growth rate and tumor weight in mouse tumor xenograft models. The tumor promoting effects of YRS was reported to depend on the PI3K/Akt signaling pathway, which was also the main pathway in YRS induced upregulation of homologous recombination (Zhang et al., 2020). YRS’s tumorigenic function in GC may be attributed to its structure. Human YRS is a homodimer during its catalytic function in protein synthesis, and contains a special C-terminal domain as compared to lower eukaryotes, archaebacteria, and prokaryotes. During inflammation, YRS is secreted and dissociated into two fragments by leukocyte elastase, and both the fragments have been reported to have a pro-inflammatory effect (Wakasugi and Schimmel, 1999). The C-terminal domain induces the production of myeloperoxidase, TNF-α, and tissue factor, while the N-terminal domain (miniYRS) interacts with CXCR1/2 receptors to influence the migration of polymorphonuclear cells (PMN), promoting monomer formation and inhibiting the dimer formation (Vo et al., 2011). MiniYRS was also reported to be induced by TNF-α, and was shown to promote angiogenesis by stimulating VEGF rather than VEGFR2 (Greenberg et al., 2008; Zeng et al., 2014). Although the dissociated YRS played a pro-tumorigenic role, another study claimed that upregulated YRS translocated from the cytosol to the nucleus to protect the DNA against oxidative stress-induced damage (Wei et al., 2014). Hence, the role of YRS in GC may depend on its localization, wherein its secreted form promotes tumor progression while the nuclear YRS played a protective role.

EPRS is overexpressed in GC and is associated with a poor prognosis. As the only bifunctional enzyme in the ARS family, EPRS activates the WNT/GSK-3β/β-catenin signaling pathway and stimulates the accumulation of β-catenin in the nucleus by directly binding to SCYL2 (SCY1-like protein 2), promoting cancer cell proliferation. EPRS inhibitors such as xanthoangelol (XA) and 4-hydroxyderricin (4-HD), not only block the WNT/GSK-3β/β-catenin signaling pathway, but also have other critical roles in mouse models, including inhibiting the *Helicobacter pylori* and alcohol-induced atrophic gastritis and tumorigenesis, and suppression of spleen mass, and alleviation of the damage to the liver, lung, gut, and spleen (Liu et al., 2021).

In 198 out of 457 cases of GC, the expression of KRS was higher in the tumor cells and TAIs(tumor-associated inflammatory cells), including macrophages/monocytes, CD4-positive T cells, and neutrophils, as compared to the normal tissues. The expression of KRS was also correlated to TNF-α expression. Moreover, KRS was associated not only with a larger tumor size, higher proliferation index of tumor cells, and lymphovascular invasion, but also with a shorter overall survival (Kim B. H. et al., 2014). It is known that TNF-α induces the secretion of KRS, and the secreted KRS attracts macrophages and peripheral blood mononuclear cells, which further enhance the production of TNF-α leading to the formation of a positive feedback loop (Park et al., 2005). The close relationship between KRS and TNF-α indicates that KRS may also serve as a cytokine during the onset and development of GC.

### Colorectal cancer

According to the most recent cancer statistics, colorectal cancer is the second-most common cause of cancer death in men and women combined, and it is estimated that 1/3rd of the digestive system cancers are newly diagnosed cases of colorectal cancer (Siegel et al., 2022). Although the mortality rate of colorectal cancer patients had decreased during the most recent decade (2010–2019) by about 2% per year, more efficient therapeutic targets are urgently needed due to the large population base.

IRS2 is upregulated in the colon cancer tissues than its surrounding tissues, and the knockdown of IRS2 inhibits the proliferation and clonogenic ability of RKO cells, and increases apoptosis, suggesting that IRS2 may act as an oncogene in colon cancer (Zhong et al., 2015).

KRS is reported to be upregulated in colorectal carcinoma than in healthy volunteers, and its expression is reduced after surgery, and has a prognostic potential for colorectal cancer (Suh et al., 2020). In another study, suppression of KRS was reported to cause incomplete EMT (epithelial-mesenchymal transition) phenotype and impair the formation of focal adhesions in colon cancer cells (Nam et al., 2016), which indicated that KRS might induce cell migration. Meanwhile, some previous articles reported that KRS interacted with 67LR (67-kDa laminin receptor) to promote cell migration. 67LR is upregulated in bile duct carcinoma as well as in colorectal neoplasms, and blockage of 67LR even inhibited adhesion and invasiveness of bile duct carcinoma cells (Li et al., 2009). The close relationship between 67LR and KRS can be explained as follows: After being phosphorylated by p38MAPK at the T52 residue, KRS dissociates from MSC and translocates to the cell membrane, where in KRS inhibits the degradation of 67LR, thereby promoting laminin-induced cell migration (Berno et al., 2005; Kim et al., 2012). Based on the above mechanism, KRS inhibitors were developed as anti-cancer agents. For example, the KRS inhibitor BC-K-YH16899, binds to KRS and blocks the interaction between KRS and 67LR without affecting the catalytic activity of KRS, and has been validated to have an anti-metastatic effect in mouse model of lung cancer (Kim D. G. et al., 2014). Another inhibitor SL-1910, was also reported to inhibit the migration of breast cancer cells (Lee et al., 2021). Another mechanism could also explain KRS mediated cell migration. In a study based on 3D-gels, KRS-positive colon cancer spheroids secreted GAS6 (growth arrest-specific6) by activating MiTF (microphthalmia-associated transcription factor). GAS6 promoted the polarization of M1 macrophages into M2 macrophages, and the M2 macrophages were dependent on the secretion of FGF2 (fibroblast growth factor 2)/GRO-α (rowth-regulated oncogene-α)/M-CSF (macrophage colony-stimulating factor) to activate the surrounding cells, thereby promoting cancer metastasis (Nam et al., 2018). KRS induced inflammation (Kim E. Y. et al., 2017; Rabouille, 2017), as well as it’s close relationship with TNF-α as mentioned above in GC, may be involved in colorectal cancer tumorigenesis.

LRS was reported to participate in mTOR1 activation by binding to RAGD. Given that mTOR plays a pivotal role in tumorigenesis, LRS inhibitors have been considered as an alternative treatment for cancers. The leucinol analog (S)-4-isobutyloxazolidin-2-one, inhibited LRS-mediated activation of mTORC1 and showed cytotoxic effects even against SW620 cells, which are known to be resistant to rapamycin (Yoon et al., 2016). Another LRS inhibitor BC-LI-0186, blocked the LRS–RagD interaction and inhibited the proliferation of HCT116 (human colon cancer cell line) cells (Kim J. H. et al., 2017).

As described above, WRS has been identified as an oncogenic protein in GC. Contrary to that, the low expression of WRS in colorectal cancer tissue was associated with an increased risk for recurrence and poor survival, which implied the protective role of WRS in colorectal cancer (Ghanipour et al., 2009). When the N-terminal domain of WRS was deleted, the new form of WRS called T2-TrpRS, exhibited an anti-angiogenic potential in neonatal mouse retinal model (Otani et al., 2002; Zhou et al., 2008). Ghanipour et al. (2009) assumed the anti-neoangiogenic potential of the new-formed fraction of WRS may be related to the protective role of WRS in colorectal cancer.

In an earlier study, MRS was reported to be highly expressed in colon cancer (Kushner et al., 1976). Subsequent studies showed that the increased copy number of MARS was associated with a higher risk for developing CRC (Wang et al., 2018). Meanwhile, HF (high fat) diet elevated MRS in the mouse colon, and MRS catalyzed the production of Hcy (homocysteine) (Perła-Kaján et al., 2007) and HTL (homocysteine thiolactone) (Jakubowski et al., 2000). K-Hcy (lysine Homocysteine) weakened the interaction between ATR (ataxia-telangiectasia and Rad3-related protein) and ATRIP (ATR-interacting protein) in combination with HTL, and inhibited the downstream effectors such as checkpoint kinase-1 and p53, promoted cell proliferation, and increased DNA damage under DNA damage stress. Consistently, the knockdown of MRS reversed these effects (Wang et al., 2018). Similar to K-Hcy, both Hcy and HTL showed a DNA damage promoting effect (Huang et al., 2001; Zhang et al., 2005). MRS-stimulated elevation of K-Hcy, Hcy, and HTL, and their DNA damage promoting effects which indicated that MRS might play a significant role in colon cancer onset and progression.

### Liver cancer

Liver cancer, which includes hepatocellular carcinoma (HCC), intrahepatic cholangiocarcinomas, as well as other rare types of cancers, has been shown to have the fastest growing mortality rates for decades. Glutamine exerted an anti-apoptotic effect on human hepatocellular carcinoma cell line HuH-7, and this effect was explained to arise from glutamine-dependent glutathione (GSH) supplement (Xu et al., 1997). However, another study provided a different perspective for glutamine’s anti-apoptotic function, wherein QRS was reported to inhibit ASK1 (apoptosis signal-regulating kinase1) to suppress glutamine-related apoptosis (Ko et al., 2001). ASK1 is a pivotal kinase in the TNF and Fas signaling pathway that induces apoptosis, and inactivation of the liver-specific ASK1 improves non-alcoholic fatty liver disease (NAFLD) (Zhang et al., 2018). It is still unclear whether QRS participates in the development of NAFLD and subsequent progression of HCC by inhibiting ASK1.

DARS2 (mitochondrial aspartyl-tRNA synthetase) was strongly upregulated in HCC, and the increase in DARS2 expression was correlated with tumor size, cell differentiation, distal metastasis, and portal vein invasion in HCC and a shorter survival time. DARS2 promoted HCC tumorigenesis by accelerating cell cycle progression and attenuating cell apoptosis. In addition, DARS2 was upregulated in HBV-infected patients and HBV infected HepG2.2.15 cells. Upregulation of DARS2 by HBV is controlled by the miR-30e-5p/MAPK/NFAT5 (Nuclear factor of activated T-cells 5) pathway. Furthermore, NFAT5 acts as a tumor suppressor in HBV-associated HCC tissues by suppressing DARS2 expression, indicating that DARS2 may be a potential target for the treatment and diagnosis of HCC (Qin et al., 2017).

A recently published study showed that YRS expression in HCC cell lines and clinical tissues was higher than in normal control samples, and that the increased expression of YRS was positively correlated with poor survival of HCC patients. Additionally, YRS is an essential component of NCK1-AS1/miR-22-3p/YRS, through which the lncRNA NCK1-AS1, positively modulates YRS, by controlling miRNA-22-3p availability and activating the PI3K/AKT pathway (Zhou et al., 2022). Certainly, such findings warrant further investigation in other tumor types to assess the function of YRS as a cancer biomarker.

GRS was reported to be upregulated in HCC and was significantly correlated with poor survival and immune cells infiltration (Wang et al., 2022). Upregulation of GRS, which is induced by histone modification of the GARS promoter, promotes HCC progression *in vivo* and *in vitro* by accelerating the cell cycle and inhibiting apoptosis. Pro-tumorigenic activity of GRS is accomplished by inhibiting the expression of pro-apoptotic proteins such as cleaved PARP, cleaved caspase3 and cleaved caspase9, and increasing the expression of pro-survival proteins including PCNA, Bcl2, Bcl-xL, and Mcl-1 (Wang et al., 2022). However, earlier studies have reported that secreted GRS from macrophages bound to cadherin 6 (CDH6), and released phosphatase 2A (PP2A) to induce apoptosis in cancer cells by suppressing ERK signaling pathway (Park et al., 2012). Other studies also showed that secreted GRS exerted anti-tumorigenic effects (Goughnour et al., 2020; Park et al., 2022). Park et al. (2022) demonstrated that secreted GRS in extracellular vesicles (EVs) interacted with different cellular receptors to function such as WHEP domain in the N-terminal interacted with CELSR2 (cadherin EGF LAG seven-pass G-type receptor 2) and activated the RAF-MEK-ERK pathway to induce M1 polarization of macrophages, while the C-terminal tRNA-binding domain bound to CDH6 to promote apoptosis. However, contrasting effects may be caused due to different localization of GRS in the cells or its secretion. Even in the secreted form, GRS plays different roles by interacting with different proteins on its different domains, indicating that further factors need to be accounted for when discussing ARSs as potential biomarkers in cancer.

### Pancreatic cancer

Pancreatic cancer is the third leading cause of cancer death in men and women combined, and is a poor-outcome disease with mortality rates nearly identical to the incidence rates. According to Jeong et al. (2018), the inhibitory effect of threonine deprivation on the migration of pancreatic cancer cells depended on MUC1 (Mucin1), a threonine-rich oncoprotein upregulated in several types of cancer, which contributed to neoplastic transformation, tumor associated angiogenesis and metastasis (Kufe, 2009). TRS (threonyl tRNA synthetase) controlled the synthesis of MUC1, and the TRS inhibitors, borrelidin (BN) and 5′-O-(N-(l-threonyl)-sulfamoyl)-adenosine (ThrAMS), also suppressed the migration of pancreatic cancer cells via MUC1. High levels of TRS, as well as MUC1, were related to poor survival outcomes in pancreatic cancer. In an earlier study, TRS secretion was stimulated by TNF-α and VEGF, which promoted the migratory and angiogenic ability of the endothelial cells (Williams et al., 2013). Taken together, TRS may be a novel therapeutic target in pancreatic cancer.

As we mentioned above, T2-TrpRS had an anti-angiogenic function. In pancreatic cell lines, hypoxia downregulated the expression of full-length WRS and upregulated T2-TrpRS (Paley et al., 2011). As the tumor microenvironment is usually hypoxic, WRS may play a protective role in pancreatic cancer. Further research is needed to reveal the unknown biological functions of WRS and explore its therapeutic potential in pancreatic cancer.

In summary, most of the ARSs have oncogenic effect in different cancers, depending on its interaction with other molecules (protein, lncRNA, miRNA) or acting as a component of a signaling pathway. The above examples illustrate the potential role of ARSs as promising diagnostic biomarkers or therapeutic target in digestive system cancers.

## Role of Aminoacyl-tRNA synthetases in hepatobiliary diseases

### Liver fibrosis and cirrhosis

Liver fibrosis and liver cirrhosis belong to the category of chronic liver diseases (CLD), which not only cause liver dysfunction, but also increase the risk for cancer. Progressive liver fibrosis might lead to cirrhosis and finally cause liver failure, and even progress to liver cancer (Wang and Friedman, 2020). However, surgery and liver transplantation are currently the only options for patients with severe liver cirrhosis due to the lack of more efficacious therapies.

GRS is secreted from several types of cells upon exposure to stress factors such as oxidative stress, radiation damage, and starvation. In the CCl4 induced hepatic damage model, the level of GRS in peripheral circulation was much higher as compared to control mice. Besides, GRS was shown to stimulate the migration of mesenchymal stem cells (MSCs) and enhance the recruitment of MSCs to the site of injury in the liver fibrosis animal model, and mitigate liver and spleen enlargements in the CCl4-induced liver damage model. A series of *in vitro* experiments demonstrated that GRS relied on its receptor CDH-6 and PI3K/Akt and/or FAK/ERK1/2 signaling pathway to exert its functions (Park et al., 2018). Moreover, CDH-6 was not detected in normal liver, whereas four out of six hepatocellular carcinoma cell lines expressed CDH-6 abundantly (Shimoyama et al., 1995). The interaction between GRS and CDH-6 in damaged tissues and cells may enable the development of a novel druggable mechanism for treating liver fibrosis. Since it is well known that liver fibrosis is a dangerous risk factor for tumorigenesis, the high expression of CDH-6 may play an important role in the development of hepatocellular carcinoma from liver fibrosis.

Previous study demonstrated that free proline concentration was higher in patients with liver cirrhosis than in non-cirrhotic patients (Kershenobich et al., 1970). Further studies also found that the bifunctional enzyme EPRS, which also possessed PRS activity, could inhibit cardiac fibrosis. EPRS not only increased the expression of collagen protein in primary cardiac fibroblasts, but also upregulated the expression of Pro-rich genes such as SULF1. The knockdown of SULF1 inhibited TGF-β mediated myofibroblast activation (Wu et al., 2020). Similarly, in the digestive system, the knockdown of EPRS in the LX2 cells (hepatic stellate cell) not only inhibited the production ECMs (collagen I, fibronectin, Snail1, a-SMA) under TGF-b1 stimulation, but also reduced the extracellular deposition and transcriptional induction of collagen-I and fibronectin. However, overexpression of the PRS domain of EPRS alone promoted the basal expression of ECM in LX2 cells, which was further up-regulated upon TGF-b1 treatment. HF (halofuginone) treatment abolished TGF-b1–mediated and EPRS-dependent collagen I and fibronectin expression, as well as SMAD2/3 phosphorylation. In CCl4 induced mouse models of liver fibrosis, EPRS knockout induced less-significant increases in fibronectin and collagen I expression without affecting laminin levels (Song et al., 2019). Additionally, in human skin fibroblast and mouse fibrosis models, inhibitors of PRS, including HF and T-3833261, suppressed TGF-β-induced fibrosis through the Smad3 axis *in vivo* (Zeplin, 2014; Shibata et al., 2017). Another PRS inhibitor T-3861174, inhibited cell proliferation by activating the GCN2-ATF4 pathway in cancer cell lines such as SK-MEL-2 (Arita et al., 2017). Therefore, further studies are needed to demonstrate the therapeutic potential of PRS in liver fibrosis and hepatocellular carcinoma.

### Bile duct stricture

Traditionally, standard Papanicolaou (Pap) staining has been widely used to distinguish benign and malignant bile duct strictures. However, MRS staining was reported to have more promising value in the diagnosis of biliary strictures based on nobiliary brushing cytology, which was performed using specimens obtained from ERCP (endoscopic retrograde cholangiography). According to Jang et al. (2021), MRS was highly expressed in bile duct cancer, and MRS immunofluorescence staining was recommended to be used as a diagnostic method due to its high sensitivity and accuracy. MRS is the initiator of translation, and the overexpression of MRS has been shown to correlate with carcinogenesis (Pavon-Eternod et al., 2013; Birch et al., 2016; Kim S. B. et al., 2017). Two opposing theories could explain this: 1. Under UV irradiation, MRS is phosphorylated at Ser662, which induces the release and nuclear translocation of AIMP3, thereby facilitating DNA damage repair (Kwon et al., 2011); 2.MRS stabilizes CDK4 (cyclin-dependent kinase4), promotes G1-to-S cell cycle transition, and enhances the tumorigenic ability of cancer cells, and this effect is much more obvious in cancers that are deficient in p16INK4a, which blocks the function of CDK4, thereby suppressing cell cycling (Musgrove et al., 2011; Kwon et al., 2018). An editorial by Singhi and Slivka (2020) suggested that the deletion of CDKN2A (promoter of p16INK4a) in bile duct specimens was attributed to the correlation of MRS with malignancy.

On the whole, previous studies on ARSs have mainly focused on the pathogenicity of ARSs, not only in cancers but also in benign diseases such as liver fibrosis and bile duct stricture. As for clinical significance, MRS immunofluorescence staining of the bile duct stricture has played a leading role in translating basic science into practical application in the clinic.

## Role of Aminoacyl-tRNA synthetases in digestive system infection

### Helicobacter pylori

*Helicobacter pylori* is the main risk factor for stomach cancer, with almost 90% of new cases of non-cardiac GC attributed to this bacterium (Bray et al., 2018). Adenosine-containing 3-arylfuran-2(5H)-ones was demonstrated as an inhibitor of YRS (Wei et al., 2017), and some of the 3-arylfuran-2(5H)-ones derivatives showed antioxidant and anti-Helicobacter pylori activities. Hence, YRS inhibitors were suggested as potential therapeutic agents against GC (Wang et al., 2015).

### HBV

WRS plays an important role not only in gastric cancer and colon cancer, but also in innate immune response. Upon bacterial infection, WRS is secreted by the monocytes, and directly binds to macrophages via a toll-like receptor 4 (TLR4)-myeloid differentiation factor 2 (MD2) complex, inducing phagocytosis and chemokine production (Ahn et al., 2016). WRS was also reported to be secreted by virus-infected immune cells in response to viral infection, and induce the secretion of pro-inflammatory cytokines and type I IFNs, resulting in the inhibition of virus replication both *in vitro* and *in vivo* (Lee et al., 2019). In a study based on LC-MS/MS analysis, WRS was validated as an important enzyme associated with HBV replication-induced angiogenesis in HepG2 cells as well as RPHs (rat primary hepatocytes) (Zhang et al., 2009). In contrast to that, another genome-wide association study showed an intergenic variant between VARS2 (valyl-tRNA synthetase2) and SFTA2 (surfactant associated2) to be associated with the risk of CHB (chronic hepatitis B) in Koreans (Cheong et al., 2015). The above studies indicated that ARSs might take part in HBV infection, HBV induced angiogenesis, and even in the broad spectrum of clinical presentations from CHB to HCC.

## Function of Aminoacyl-tRNA synthetases in autoimmune diseases

### Inflammatory bowel disease

Inflammatory bowel disease (IBD), which includes Crohn’s disease and ulcerative colitis, presents symptoms including abdominal pain, diarrhea, and intestinal obstruction. hUCB-MSCs (human umbilical cord blood-derived mesenchymal stem cells) have been considered as a promising treatment for IBD because of their anti-inflammatory effects (Kim et al., 2013). Studies have reported that the expression of WRS was downregulated in DSS-induced experimental colitis model, wherein the addition of hUCB-MSCs not only alleviated DSS-induced colitis but also upregulated the expression of WRS. It was also shown that WRS inhibited the proliferation of hUCB-MSCs derived CD4+T cells by promoting apoptosis, which indicated that WRS might have a therapeutic effect in autoimmune diseases by regulating the hyper-activation of regulatory CD4+ T cells (Kang et al., 2019).

### Anti-synthetase syndrome

Anti-synthetase syndrome is an autoimmune disease characterized by symptoms such as interstitial lung disease, myositis, and arthritis. AAS also includes other characteristics of connective tissue diseases, such as Raynaud’s phenomenon or gastroesophageal reflux (Rasendrakumar et al., 2022). A group of ARSs-associated antibodies, including anti-Jo-1 (anti-HRS), anti-PL7 (anti-TRS), anti-PL12 (anti-AlaRS), anti-OJ (anti-IRS), anti-EJ (anti-GRS), and other less common antibodies, are regarded as a hallmark of ASS (Witt et al., 2016). Cancer-associated AAS was reported in colon adenocarcinoma and lung cancer (Rozelle et al., 2008; Zang et al., 2008), and cancer treatment alleviated some of the symptoms of AAS. Moreover, a case report by Phillips et al. (2022) demonstrated that an increase in the incidence of ASS coincided with COVID-19. Due to various clinical presentations of ASS that may overlap with some of the symptoms of COVID-19 and the paraneoplastic syndrome of certain cancers, some researchers think it is reasonable to suspect that the diagnoses of ASS may be underestimated.

Previous findings indicated that ARSs were involved in the maturation, activation, and recruitment of immune cells, thus playing a crucial role in the development of immune cells (Park et al.,. 2005; Noh et al., 2015; Jobin et al., 2019; Nie et al., 2019). In this review, we discussed the role of ARSs as regulators and signaling molecules in infectious diseases and as autoantigens in autoimmune diseases. However, further in-depth studies are needed to explore the underlying molecular mechanisms associated with the specific disease.

## Others

### Gastrointestinal stromal tumors

Based on the analysis of tissue microarrays, IDO and WRS were associated with tumor size, mitosis, and outcomes in GIST patients. In 127 pairs of GIST located in the gastric, small intestine, and other tissues, 114 were positive for IDO (89.8%) expression, while 60 were positive for WRS (47.2%) (Blakely et al., 2018). This may be consistent with the balance between IDO and WRS that we mentioned before.

### ARSs-mutation related digestive system diseases

Mutations in ARSs exhibit a close relationship with some inherited diseases in humans, which makes patients more susceptible to digestive system related phenotypes such as elevated liver enzymes and hepatomegaly (Prasun et al., 2019), liver steatosis (Kuo et al., 2019), liver cirrhosis (Xu et al., 2018), and advanced liver disease associated portal hypertension (Peluso et al., 2021). MARS gene mutation has been shown to cause ILLD (Interstitial lung and liver disease), which includes several liver abnormalities such as hepatomegaly, cholestasis, hepatic steatosis, fibrosis, and iron deposition (Abuduxikuer et al., 2018). Frameshift mutation in MARS gene was also found to be associated with gastric and colorectal carcinomas with microsatellite instability (Park S. W. et al., 2010). In a recently published study, recessive mutation in IARS was validated as a novel monogenic cause of IBD, which caused persistent pancolitis and resistantance to mesalazine, corticosteroids, azathioprine, sirolimus and anti-TNF (adalimumab) therapeutics (Fagbemi et al., 2020). ILFS1 (infantile liver failure syndrome type1), resulting from LARS mutation, appeared with clinical symptoms including low birth weight, early failure to thrive, anemia, hypoalbuminemia, and liver dysfunction before the age 1, further expanding the phenotype of ARSs related hereditary diseases (Casey et al., 2015).

## Conclusion

Astronomical amounts of research funding and efforts have been invested into the non-catalytic role of ARSs in various kinds of diseases. Herein, we describe the pathology of ARSs in digestive system diseases, especially in malignancies of the organs of the digestive system. Indeed, alteration in the expression and intracellular localization, mutation, molecular interaction, secretion or genetic variants of ARSs not only stimulates or dampens the clinicopathological development and survival of cancerous cells, but also influences a set of phenotypes, such as invasion, migration, proliferation, cell cycle regulation, DNA damage repair, and others. Alteration in the expression level, copy number variation, and secretion of ARSs may serve as prognostic biomarkers, while its mutation, molecular interaction, and altered localization may serve as promising targets for cancer therapy.

ARSs are essential enzymes for protein biosynthesis in both normal cells and cancerous cells. Upregulated ARSs in cancer cells not only play catalytic role to meet the increased demand for protein synthesis of cancer cells for growth, but also have rich connections with multiple of tumourgenic processes. First, ARSs involved in a broad spectrum of cellular signaling pathways by interacting with diverse cellular factors, which may happen in different domain from the domain that play catalytic role, such as GRS in HCC (Wang et al., 2022),. Secondly, some of ARSs have special ability in sensing intracellular amino acid level in an amino acid-dependent manner, thus take part in signaling pathways and manitain homeostasis of amino acid pools and cellular metabolome, such as LRS (Lee et al., 2018). Besides, in certain circumstance, ARSs can be cut into spieces, wich have new feature in tumourenisis, such as WRS in pancreatic cancer (Paley et al., 2011). Moreover, change in cellular localization and secretion of ARSs in cancer cell exert unique activities during the development of cancers, such as YRS in gastric cancer (Wei et al., 2014), and KRS in colon cancer (Nam et al., 2018). As persuasive examples disolayed above, the relations between cancer and ARSs have inspired the development of innovative cancer treatments that target or take advantage of these novel functions of ARSs.

Currently, anti-infective agents targeting ARSs have attracted a significant amount of attention. Several ARSs inhibitors have been applied in the clinical practice including the IRS inhibitor anti-bacterial mupirocin, for the treatment of topical infections caused by G-positive bacteria (Pappa, 1990; Nakama et al., 2001), the LRS inhibitor anti-fungal AN2690, for the treatment of onychomycosis which is caused by dermatophytes (Rock et al., 2007), and the PRS inhibitor anti-protozoal halofuginone, for the treatment of malaria (Samant and Sukhthankar, 2009). Moreover, the YRS inhibitor ML901, exhibits whole-life-cycle killing activity in a mouse model of malaria (Xie et al., 2022), and the LRS inhibitor GSK656, has been used in clinical trials for systemic use against tuberculosis (Tenero et al., 2019). Since ARSs are essential enzymes both in humans and pathogens such as bacteria, fungus, and parasites, it is important for antimicrobial drugs to exhibit toxicity against the targeted organism while leave the human physiology unaffected. This fundamental requirement of anti-infective drugs targeting ARSs can be fulfilled due to their diverse biological structures across different species or differences in the sequence and topology of ARSs. For example, human LRS and bacterial LRS display significant variability in the editing site. The human LRS has lost editing site during evolution (Lue and Kelley, 2005). The LRS2 inhibitor GSK656, which demonstrated anti-tuberculosis activity, is high selective for the *Mycobacterium tuberculosis* LRS due to its special design that trapped the three end of tRNA^Leu^ in the editing site, inhibiting leucylation and thus protein synthesis (Li et al., 2017). Another study clarified that the high selectivity of the anti-fungal LRS inhibitor AN2690, could be attributed to its inefficient penetration into the human cells and its inability to access MSC (Yao and Fox, 2013). Besides, normal cells have the ability to withstand significant suppression of ARSs with little effect on global translation, which makes the PRS inhibitor halofuginone, an attractive therapeutic agent (Kwon et al., 2019). Table 2 summarizes the ARSs inhibitors in the digestive system.

**TABLE 2:** ARSs related inhibitors and their effect in the digestive system.

ARSs	Inhibitor(s)	Related disease	Function	*In vitro*/*vivo*	References
EPRS	XA,4-HD	Gastric cancer	Block WNT/GSK-3β/β-catenin signaling pathway	*in vitro*	Liu et al. (2021)
			Hinder the Hp and alcohol-induced atrophic gastritis	*in vivo* (mouse)	
			Inhibit t Hp combined with alcohol-induced gastric tumorigenesis		
			Decrease the weight of the spleen		
			Alleviated damage to the liver, lung, gut, and spleen		
LRS	BC-LI-0186	Colon cancer	Blocks the LRS–RagD interaction	*in vitro*	Kim J. H. et al. (2017)
			Inhibits the proliferation of HCT116		
	(S)-4-isobutyloxazolidin-2-one	Colon cancer	Inhibits LRS-mediated activation of mTORC1	*in vitro*	Yoon et al. (2016)
			Shows cytotoxicity to rapamycin-resistant colon cancer cells SW620		
TRS	BN,ThrAMS	Pancreatic cancer	Suppresses the migration of pancreatic cancer	*in vitro*	Jeong et al. (2018)
YRS	3-arylfuran-2(5H)-ones derivatives	Gastric ulcer	Antioxidant and anti-Hp	*in vitro*	Wang et al. (2015)
PRS	HF(halofuginone)	Liver fibrosis	Abolish collagen I and fibronectin expression, inhibiits SMAD2/3 phosphorylation	*in vitro*	Song et al. (2019)

Among the exciting questions for the future, one of the most vital one is whether ARSs could serve as anti-cancer therapeutics and influence tumor development and progression. However, in non-infectious conditions such as cancer, which have a high demand for protein synthesis, the development of drugs targeting ARSs requires further in-depth exploration. Inhibitors targeting ARSs may be engineered to target their special motifs, and their close correlation with proteins from signaling pathways proteins, or their disease-associated protein-protein interactions. Further research is warranted to demonstrate the therapeutic potential of ARSs in treating digestive system diseases.

## Abbreviations

ARSs, aminoacyl-tRNA synthetases; AlaRS, alanyl-tRNA synthetase; CRS, cysteinyl-tRNA synthetase; DRS, aspartyl-tRNA synthetase; ERS, glutamyl-tRNA synthetase; EPRS, glutamyl-prolyl-tRNA synthetase; FRS, phenylalanyl-tRNA synthetase; GRS, glycyl-tRNA synthetase; HRS, histidyl-tRNA synthetase; IRS, isoleucyl-tRNA synthetase; KRS, lysyl-tRNA synthetase; LRS, leucyl-tRNA synthetase; MRS, methionyl-tRNA synthetase; NRS, asparaginyl-tRNA synthetase; PRS, prolyl-tRNA synthetase; QRS, glutaminyl-tRNA synthetase; RRS, arginyl-tRNA synthetase; SRS, seryl-tRNA synthetase; TRS, threonyl-tRNA synthetas; VRS, valyl-tRNA synthetase; WRS, tryptophanyl-tRNA synthetase; YRS, tyrosyl-tRNA synthetase.
